# Supplementary material for: Estimating spatiotemporally varying malaria reproduction numbers in a near elimination setting
Source: Nat Commun. 2018 Jun 26;9:2476. doi: 10.1038/s41467-018-04577-y (PMC6018772; doi:10.1038/s41467-018-04577-y)
Supplement: Supplementary file 1 — Supplementary Information [file 41467_2018_4577_MOESM1_ESM.pdf]

Supplementary Information

Estimating spatiotemporally varying malaria reproduction numbers in a near elimination setting

Routledge *et al*.

Contents

- **Supplementary Figure 1:** Symptom to treatment time
- **Supplementary Figure 2:** Marginal gain in likelihood when adding edges to transmission tree from sampled serial intervals
- **Supplementary Figure 3:** Sensitivity analysis
- **Supplementary Figure 4:** AUC analysis
- **Supplementary Table 1:** Contextual details for El Salvador
- **Supplementary Table 2:** covariates used in risk mapping R_c>1
- **Supplementary Note 1:** Georeferencing cases
- **Supplementary Note 2:** Sensitivity analysis
- **Supplementary Note 3:** Derivation of methods for determining the most likely transmission trees and R_c
- **Supplementary Note 4**: Mapping $R_c\ (t)$
    - Covariate assembly
    - Spatial methodology

Supplementary Figure 1: Symptom to treatment time

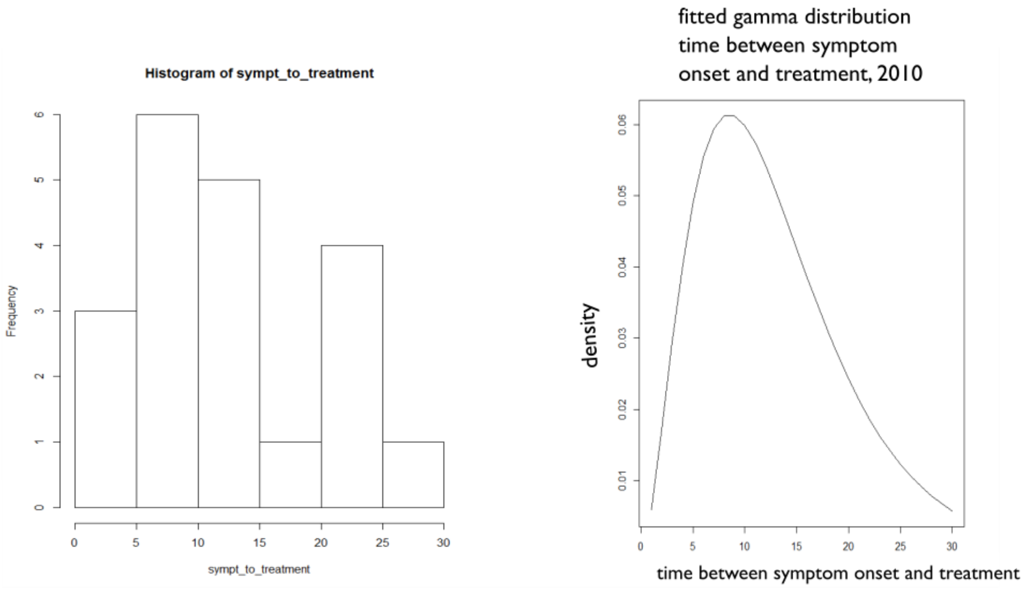

Distribution of time from symptoms to treatment, based on data from year 2010. Raw data as histogram and fitted gamma distribution shown.

Supplementary Figure 2: Marginal gain in likelihood when adding edges to transmission tree from sampled serial intervals

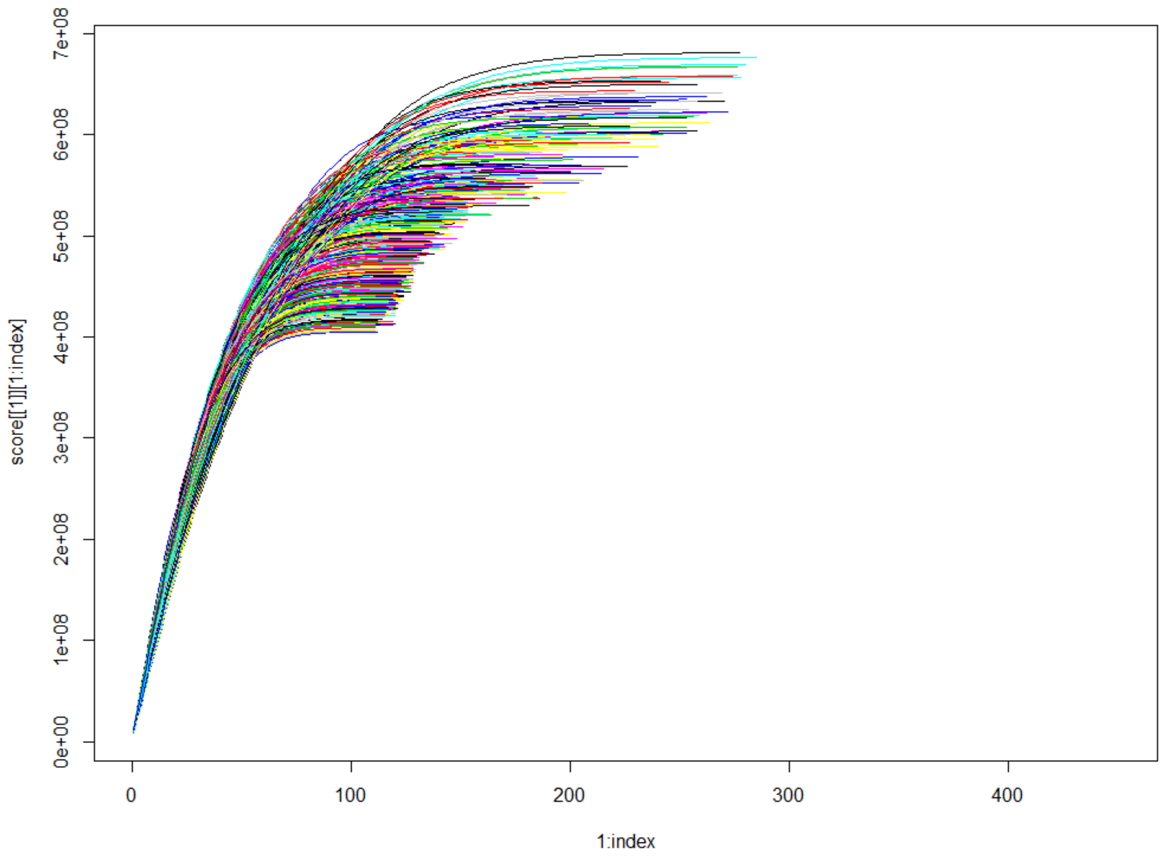

Marginal gain in overall likelihood of transmission route when adding extra links between nodes. Point where marginal gain <=1e-5 is point where we stop drawing edges.

Supplementary Figure 3: Sensitivity analysis

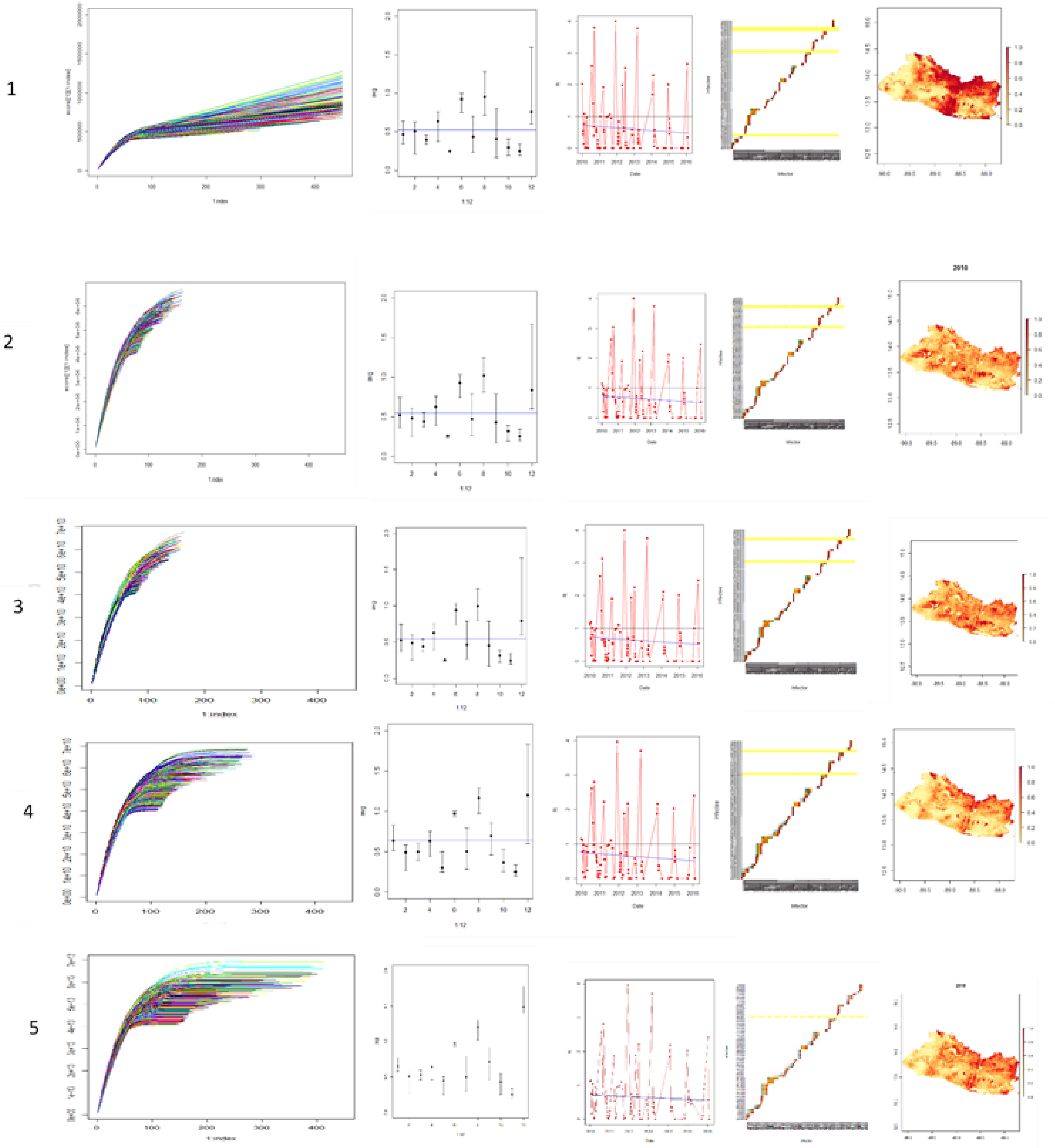

Scenario 1: epsilon = 0.01 and tolerance for edges = 0.003, Scenario 2: epsilon = 0.003 and tolerance for edges = 0.003, Scenario 3: epsilon = 0.007 and tolerance for edges = 0.003, Scenario 4: epsilon = 0.007 and tolerance for edges = 0.005, Scenario 5: epsilon= 1e-10, tolerance for edges = 1e-10

Supplementary Figure 4: AUC scores for map (AUC = 0.94)

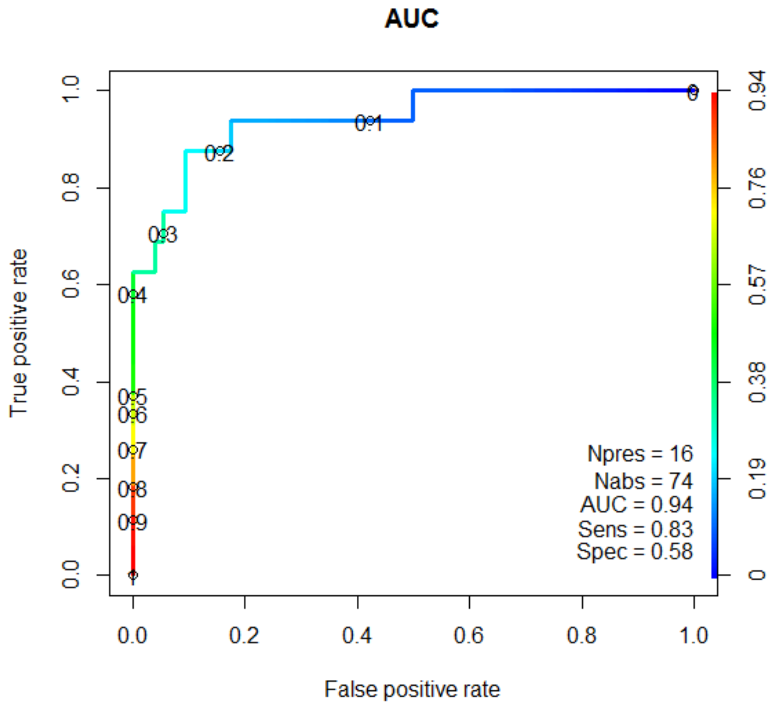

Supplementary Table 1: Contextual details for El Salvador

Slides examined per year[1]:

Year	Slides examined
2010	115 000
2011	100 883
2012	124 885
2013	103 748

Supplementary Table 2: covariates used in risk mapping R_c>1

variable class	variable(s) source type		
**temperature**	land surface temperature (day, night and diurnal-flux)	MODIS product	dynamic monthly
**temperature suitability**	temperature suitability for *P. vivax*	modelled product	dynamic monthly
**precipitation**	mean annual precipitation	WorldClim	synoptic
**vegetation vigour**	enhanced vegetation index	MODIS derivative	dynamic monthly
**surface wetness**	tasselled cap wetness	MODIS derivative	dynamic monthly
**surface brightness**	tasselled cap brightness	MODIS derivative	dynamic monthly
**IGBP landcover**	fractional landcover	MODIS product	dynamic annual
**IGBP landcover pattern**	landcover patterns	MODIS derivative	dynamic annual
**terrain steepness**	SRTM derivatives	MODIS product	static
**flow & topographic wetness**	topographically redistributed water	SRTM derivatives	static
**elevation**	digital elevation model	SRTM	static
**human population**	WorldPop	modelled products	dynamic annual
**infrastructural development**	accessibility to urban centres and night-time lights	modelled product and VIIRS	static
**moisture metrics**	aridity and potential evapotranspiration	modelled products	synoptic

Supplementary Note 1: Georeferencing cases

All cases within El Salvador with full addresses were georeferenced to caserío/ lotificación level, which is approximately neighbourhood or hamlet level.

Name searches of streets, caseríos, and landmarks were carried out using Nominatim on Open Street Map[2]. Google and Bing maps[3,4] were also used to cross check and in the absence of information available on open street map. We also used several locality listing websites[5–9] to obtain and/or cross check georeferences for caseríos.

Municipality (municipio) and district (distrito) were also provided, allowing cross checking for duplicate neighbourhood names and ensure continuity. In addition, searches were made online for local schools, churches, news stories and community groups to cross check locations. Many addresses listed geographic features such as landmarks or road names. Where possible, Google satellite imagery were examined for these features and/or evidence of dwellings.

Supplementary Note 2: Sensitivity analysis

We explored the sensitivity of our approach by varying the threshold likelihood for linking cases, ϵ, and the threshold gain in marginal likelihood used to define the number of edges to create, K. We consider several scenarios, illustrated below:

Scenario 1: epsilon = 0.01 and tolerance for edges = 0.003

Scenario 2: epsilon = 0.003 and tolerance for edges = 0.003

Scenario 3: epsilon = 0.007 and tolerance for edges = 0.003

Scenario 4: epsilon = 0.007 and tolerance for edges = 0.005

Scenario 5: epsilon= 1e-10, tolerance for edges = 1e-10

Supplementary Note 3: Derivation of methods to determine the most likely transmission trees and R_c

We assume cases were classified correctly from case investigation as imported or locally-acquired based on recent travel history. Following this assumption, locally acquired cases could have both infected others and been infected themselves. Imported cases could only infect others, as we assume their infection was acquired outside of the country. We also assume a case showing symptoms at time t has been infected by a case which began showing symptoms earlier in time, due to the short time between symptom onset, presentation at health facilities and the beginning of treatment.

To infer the most likely pathway of transmission linking observed cases, we use a modified version of [10] based upon the independent cascade model [11]. Out data input consisted on a time series of symptom (fever) onset $t \in \{t_1, \ldots, t_n\}$, time ordered such that $t_1 < t_2, \ldots, < t_n$. While the times of symptom onset are known, what is not known is who infected whom. The goal of our model is to infer the most probable network structure, $\mathcal{G}$, connecting these n infections. We infer $\mathcal{G}$ solely from the symptom onset times t, a serial interval distribution, and hyperprior probability distributions for the serial interval distribution parameters. The serial interval is the time between a given case, j, showing symptoms and the appearance of symptoms in a case i infected by the earlier case, such that $t_j < t_i$ [12]. The serial interval distribution specifies a normalised pairwise transmission likelihood, or the likelihood of case j infecting case i, given the time between symptom onsets, $t_i - t_j$.

We defined a range of priors for possible serial interval distributions to allow for possible variation in transmission dynamics due, for example, to imported infections with *P. falciparum*. Six cases imported to El Salvador were *P. falciparum*, however, the remainder of cases were *P. vivax*. The serial interval distribution of treated, symptomatic *P. falciparum* malaria, previously characterised using empirical and model-based evidence [13] was adapted to inform the prior distribution for the relationship between time and likelihood of transmission between cases in El Salvador. As a result the prior distribution was altered to better reflect the biology of *P. vivax* and the dominant vector species in El Salvador, *Anopheles albimanus,* but was sufficiently uninformative to allow for possible variation in

transmission dynamics. In addition, there is a possibility of a small number of asymptomatic or undetected and therefore untreated infections contributing to ongoing transmission, which will take on a longer serial interval. By defining a prior for the shape parameter of a serial interval distribution we can account for some of this uncertainty.

The serial interval can be thought of as consisting of: a) time between symptom onset and infectiousness onset[1] b) the time for humans to transmit malaria parasites to mosquito vectors[2] (c) the period of mosquito infectiousness, and d) the human incubation period. Following recent work in a variety of contexts (currently under review) suggesting that the initial blood stage of infection for *P. vivax* is shorter than *P. falciparum* (24 and 29 days compared to 33 and 136 days respectively for *P. falciparum*), the expected human to mosquito transmission time was made shorter than the expected value from the distribution for *P. falciparum* in Huber et al[13].

We use a shifted Rayleigh distribution with the form: $f(t_i|t_j;\alpha,\gamma) = \alpha(t_i - t_j - \gamma)e^{-\alpha(t_i-t_j-\gamma)}$, where α governs the shape of the distribution and γ is the shifting parameter accounting for the incubation period between receiving an infectious bite and the onset of symptoms (see Figure 1A). The hyperprior for the α parameter determining the shape of the Rayleigh distribution was a uniform prior, bounded between 0.001 and 0.01, giving an expected value of 0.005. This gives an expected time between symptom onset of one case and symptom onset of the case it infects of 29 days (95%CI = 16 – 300 days, sd = +/- 7 days (23-37)), with the lower bound of 25 days (95%CI =16 – 299 days, sd = +/- 4 days(21-28)) and the upper 47 days (95%CI = 16-300 days sd= +/- 18 days (30-66)). By comparison the expected values for treated P. falciparum from existing literature range between 33

---

[1] Symptoms such as fever are triggered by the rupture of merozoites infected by asexual parasites, infectiousness is determined by the presence of male and female gametocytes in the blood. We assume this time difference to be negligible, however malaria therapy data found that fever was observed just before the first gametocytes were observed but 4 days before both male and female gametocytes were simultaneously observed.

[2] LEP was fixed at 6 days – supported to be same for *P. vivax*. Simulations[23] of the within-host dynamics of *P. vivax* estimate the mean days to detectable parasitaemia was six days after the release of blood-stage parasites from the liver (95% confidence intervals 3.6-8.4 days).

days [14] and 49.1 days (95%CI = 33- 69) [13]. The hyperprior for the γ shifting parameter was set between 10 and 15 days to account for the extrinsic incubation period within the mosquito and the time between infection and suitable numbers of gametocytes in the blood to lead to symptom onset.

There were no confirmed relapse cases in the dataset and all cases were treated with Primaquine and Chloroquine (radical cure) after being detected (treatment is initiated before cases are confirmed by microscopy). Given this, we assume that a case can only be infected once by a case which has shown symptoms earlier in time. For a possible transmission tree $\mathcal{T}$ connecting cases with a set of transmission events or edges linking cases, $\mathcal{E}_{\mathcal{T}}$, the likelihood of observing symptom onset times conditional on a given $\mathcal{T}$ is: $f(t|\mathcal{T}) \propto \prod_{(u,v)\in\mathcal{E}_{\mathcal{T}}} f(t_u|t_v;\alpha,\gamma)$. Given this likelihood on a single transmission pathway $\mathcal{T}$, the underlying graph is found by considering all possible transmission pathways supported by a given network $\mathcal{G}$: $f(t|G) \propto \sum_{\mathcal{T}\in T(\mathcal{G})} f(t|\mathcal{T})\mathbb{P}(\mathcal{T}|\mathcal{G})$ where $T(\mathcal{G})$ is the set of all the possible transmission pathways for $\mathcal{G}$. By imposing a flat prior on $\mathbb{P}(\mathcal{T}|\mathcal{G})$ and as a consequence of the assumptions of a single parent node with an earlier symptom onset date our likelihood simplifies to

$$f(t|\mathcal{G}) \propto \sum_{\mathcal{T}\in T(\mathcal{G})} \prod_{(u,v)\in\mathcal{E}_{\mathcal{T}}} f(t_u|t_v;\alpha,\gamma) \qquad (1)$$

Our derivation until this point is the same as that introduced by Wallinga and Teunis [15] and extended to a wide variety of contexts by others [16]. However, methods based on Wallinga and Teunis make the strong simplifying assumption that the likelihoods of all spanning trees on $\mathcal{T}$ and therefore $\mathcal{G}$ are constant. Thus they fundamentally infer the most probable underlying network structure or jointly consider all infection times at once. In contrast, by following the approach introduced by Gomez-Rodriguez and Shölkopf [17], we solve the optimisation problem $G = \max_{|G|\le k} f(t|G)$ for a set of at most k edges, or transmission events linking cases. The two fundamental challenges with solving this optimisation problem are (a) the sum $\sum_{\mathcal{T}\in T(\mathcal{G})}(\cdot)$ is evaluated over all directed spanning trees in $\mathcal{G}$, which can be super-exponential in n, and (b) $\max_{|G|\le k} f(t|G)$ is a special case of the maximum coverage problem which has been proven to be NP-hard [18] and therefore unsolvable without searching a

combinatorial graph space with brute force. Following previous approaches [17,19], challenge (a) can be solved by observing that the resulting matrix $f(t_u|t_v;\alpha,\gamma)$ for all $(u,v)\in\mathcal{E}_{\mathcal{T}}$ pairs is an upper triangular connectivity matrix. From Tutte and Gomez and Shölkopf[10] the connectivity matrix can be expressed as a determinant, which for an upper triangular matrix is the product of the diagonal elements. Therefore the likelihood in equation (1) becomes tractable and can be evaluated in quadratic time as:

$$f(t|\mathcal{G}) \propto \prod_{t_i\in t} \sum_{t_j\in t, t_j<t_i} f(t_i|t_j;\alpha,\gamma) \qquad (2)$$

Equation (2) is better evaluated on a log scale $F(t|\mathcal{G}) \propto \sum_{t_i\in t} \log\left(\sum_{t_j\in t^c: t_j\le t} f(t_i|t_j;\alpha,\gamma)\right)$

For challenge (b) it can be proved[17] that, while finding an optimum to solve $\max_{|G|\le k} F(t|G)$ is NP-hard, the structure of $F(t|G)$, namely $F: 2^W \longrightarrow \mathbb{R}$ power set mappings for a finite set W to $\mathbb{R}$, is submodular. Submodularity in the structure of $F(t|G)$ yields a natural property of diminishing returns. That is, the incremental value that a single edge makes when added to $\mathcal{G}$ decreases as the size of the graph increases. Optimising submodular functions is possible using the greedy algorithm with provable and near-optimal performance guarantees [20] [11]. To implement the greedy algorithm we start with an empty graph, $\mathcal{K}$, and then add edges sequentially such that the *marginal gain* from each iteration is maximised. Formally, this means we start with $\mathcal{G}=\mathcal{K}$ a and then each iteration (m) evaluate the edge $e_m \in \{i,j\}\ \forall j<i$ that yields the best marginal gain, $e_m = \max_{e\in\mathcal{G}\setminus\mathcal{G}_{m-1}} F(\mathcal{G}_{m-1}\cup\{e\}) - F(G_{m-1})$, and add this edge to our graph $\mathcal{G}=\mathcal{G}\cup\{e_m\}$. We stop when we have reached $\mathcal{G}=\{e_1,\dots,e_k\}$ edges. Due to submodularity, the solution quality on increases with each additional edge, however, the marginal gain quickly asymptotes, thus ensuring sparse solutions. We stopped adding additional edges when the marginal gain in likelihood of adding edges fell below 0.0001(see figure S1).

We make two modifications to the above optimisation algorithm. Firstly, to incorporate edges known to be importations, we constrain child/infectee, i, edges in $e\in\{i,j\}\forall j<i$ to be only non-imported infections. This ensures that local infections $cannot$ infect imported infections, but imported

infections can infect any node. Secondly, to account for variation in the serial interval distribution, we run the above greedy scheme for prior samples of α, γ, as discussed above.

Our approach naturally lends itself to Bayesian formulations. As it currently stands our formulation uses a proportional likelihood optimised by exploiting submodularity. However equivalent frameworks exist [21]with explicit likelihoods derived from a survival view point of a temporal point process. These methods can be used within a full Bayesian hierarchical model.

Accounting for missing cases

Assuming all cases reaching community health workers or health facilities are recorded, missing cases may be generated by two processes. Symptomatic cases may be missed by not seeking care or being found through active case detection. On the other hand, cases may be asymptomatic and therefore unlikely to seek care or be detected. They may have densities of parasites in their blood which are too low to be detectable by microscopy if active case detection occurs. These reasons for missed detection apply to both imported cases and locally acquired cases. We assume the pool of asymptomatic cases in the country is low and has a small contribution to ongoing transmission. To explore the amount of cases which may be going undetected within our independent cascade framework, we consider additional edges π, that represent unobserved individuals who can infect any observed individual, i, in a transmission chain. Every observed individual i can get infected by unobserved individuals, π, with an arbitrarily small probability ε. This so called ϵ-edge is connected to every node in our network and do not, by design, participate in the diffusion propagation. The ϵ-edges prevents breaks in the network diffusion cascade where the likelihood of transmission between observed cases is sufficiently low, the case is linked to an external source. Additionally ϵ-edges ensure the likelihood is monotonic, that is, converting an ϵ-edge to a network edge in $\mathcal{G}$ only increases the likelihood. The addition of ϵ-edges was achieved by augmenting our pairwise transmission likelihood as follows: $f(t_i|t_j;\alpha,\gamma) = \epsilon^{-1}\alpha(t_i - t_j - \gamma)e^{-\alpha(t_i-t_j-\gamma)}$

The specific value of ε was set at 1e-5 to balance between false positives and false negatives when linking cases by infection events. The higher the value of ε, the larger the number of nodes that are assumed to be infected by an external source.

Estimating $R_c(t)$

In solving $G = \max_{|G|\leq k} F(t|G)$ via the greedy algorithm we estimate k edges $e_m \in \{i,j\}\forall j < i$ by iteratively maximising the marginal gain in the log transmission likelihood of that edge over all other edges $e_m = \max_{e\in\mathcal{G}\setminus\mathcal{G}_{m-1}} F(\mathcal{G}_{m-1} \cup \{e\}) - F(G_{m-1})$. We therefore can calculate a $(n-q) \times n$ matrix, $\mathcal{M}$, for n total infections and q imported infections of $k \leq (n-q) \times n$ marginal gains edges. The rows of the upper triangular matrix $\mathcal{M}$ are therefore the infectees and the columns the infectors. Because the solution is a positive and monotonically increasing function and $F(t|G)$ is submodular, these marginal edge gains asymptote (figure S1) thereby creating sparse solutions and diminishing gains for each additional edge.

By normalising the rows/infectees of $\mathcal{M}$ and creating a normalised matrix $\mathcal{R} = \mathcal{M}_{[i,\cdot j]} / \sum_{j=1}^{n} \mathcal{M}_{[i,j]} \,\forall\, \{i = 1,..,(n-q)\}$ we get a matrix that represents both which infector edges are connected to infectees and the normalised marginal gain of that edge. Intuitively then, by taking the row sums of $\mathcal{R}$ we get the (fractional) number of secondary infections and therefore a point estimate of the time varying reproductive number $\mathcal{R}_c(t_j) = \sum_{j=1}^{n-q} \mathcal{R}_{[\cdot,j]}$. This reflects for an individual, how many people they are likely to have gone onto infect. When multiple individuals have been infected at a given time and/or place, we can take the mean individual $\mathcal{R}_c$ and uncertainty in this value as an indicator of reproductive numbers for a given time and/or location.

Supplementary Note 4: Mapping $R_c(t)$

Covariate assembly

The environmental covariates (i.e., independent variables) used in the spatial mapping of R_c>1 risk consisted of raster layers that spanned El Salvador 2.5 arc-minute (~5 km x 5 km) spatial resolution. Covariate choice was based on key variables used within past malaria mapping endeavours[22]. Raster datasets were then acquired or produced, and wherever possible dynamic versions (i.e., temporally varying products) were utilized to support the temporal aspect of the analysis. The majority of the raster covariates were derived from high temporal resolution satellite images and then aggregated to create dynamic covariates for every month throughout the study period (2010-2016). The covariates used are listed below in Supplementary Table 2.

Spatial methodology

The underlying spatial statistical model was fitted to binomial data of $R_c > 1 = 1; R_c < 1 = 0$, using the logit link function:

$$R^{+}_{>1,i} \sim Binomial(p_i, N_i)$$

$$log(p_i/(1-p_i)) \sim GP(\mu, Q)$$

$$\mu = \alpha + X_i\beta$$

$$Q = \boldsymbol{K}_{space}$$

$$\boldsymbol{K}^{-1}_{space} = solve\ (k^2 - \Delta)^{\frac{\alpha}{2}}\big(\tau x(s)\big) = W(s)$$

where $R_{>1,i}$ are the number binary data points for $R_c > 1 = 1; R_c < 1 = 0$, p_i is the estimated $R_{>1}$, expressed as a logit transformed probability and modelled as a Gaussian process with μ and precision Q. The GP mean μ is a linear function of a global intercept α and space-time indexed

covariate values $\boldsymbol{X}_i$. Q is a sparse precision matrix constructed from a spatial process $\boldsymbol{K}_{space}$. $\boldsymbol{K}_{space}$ is the sparse finite element solution to the stochastic partial differential equation $(k^2 - \Delta)^{\frac{\alpha}{2}}(\tau x(s)) = W(s)$, where Δ is the Laplacian, k is the spatial scale/range parameter, τ controls the variance, α is the spatial smoothness parameter (fixed at $\alpha = 2$), and $W(s)$ is the spatial white noise process. To account for the curvature of the earth the distance metric s is defined on a spherical manifold in Cartesian $\mathbb{R}^3$. A sensitivity analysis showed our analysis was robust to prior specifications.

References

1. Schneider, K. et al. Malaria Elimination in El Salvador: A Historical and Epidemiological Perspective. (2016).

2. OpenStreetMap Nominatim: Search. Available at: https://nominatim.openstreetmap.org/. (Accessed: 13th March 2018)

3. Google Maps. Available at: https://www.google.co.uk/maps/. (Accessed: 13th March 2018)

4. Bing Maps - Directions, trip planning, traffic cameras & more. Available at: https://www.bing.com/maps. (Accessed: 13th March 2018)

5. Categoría:Cantones, caseríos y comunidades de El Salvador - Wikipedia, la enciclopedia libre. Available at: https://es.wikipedia.org/wiki/Categoría:Cantones,_caseríos_y_comunidades_de_El_Salvador. (Accessed: 13th March 2018)

6. El Salvador Map: Ahuachapan — Usulutan | El Salvador Google Satellite Maps. Available at: http://www.maplandia.com/el-salvador/. (Accessed: 13th March 2018)

7. Mapa e imagen satelital / imagen de satélite de El Salvador, Coordenadas GPS, Cartografía, ... Available at: http://mapasamerica.dices.net/elsalvador/. (Accessed: 13th March 2018)

8. Geographical Names List El Salvador - Geographical Names, map, geographic coordinates. Available at: https://geographic.org/geographic_names/el_salvador/index.html#F. (Accessed: 13th March 2018)

9. El Salvador map: El Salvador global, online google map. Available at: http://www.mapmonde.org/central-america/el-salvador/. (Accessed: 13th March 2018)

10. Rodriguez, M. G. & Schölkopf, B. Submodular Inference of Diffusion Networks from Multiple Trees. Icml 489–496 (2012).

11. Kempe, D., Kleinberg, J. & Tardos, É. Maximizing the spread of influence through a social network. in Proceedings of the ninth ACM SIGKDD international conference on Knowledge discovery and data mining - KDD '03 137 (ACM Press, 2003). doi:10.1145/956750.956769

12. Fine, P. E. M. The interval between successive cases of an infectious disease. Am. J. Epidemiol. **158,** 1039–47 (2003).

13. Huber, J. H., Johnston, G. L., Greenhouse, B., Smith, D. L. & Perkins, T. A. Quantitative, model-based estimates of variability in the serial interval of Plasmodium falciparum malaria. bioRxiv (2016).

14. Churcher, T. S. et al. Measuring the path toward malaria elimination. Science (80-.). **344,** (2014).

15. Wallinga, J. & Teunis, P. Different epidemic curves for severe acute respiratory syndrome reveal similar impacts of control measures. Am. J. Epidemiol. **160,** 509–516 (2004).

16. Morelli, M. J. et al. A Bayesian Inference Framework to Reconstruct Transmission Trees Using Epidemiological and Genetic Data. PLoS Comput. Biol. **8,** e1002768 (2012).

17. Rodriguez, M. G. & Schölkopf, B. Submodular Inference of Diffusion Networks from Multiple Trees. Icml 489–496 (2012).

18. Khuller, S., Moss, A. & Naor, J. The budgeted maximum coverage problem. Inf. Process. Lett. (1999).

19. Gomez-Rodriguez, M., Leskovec, J. & Krause, A. Inferring networks of diffusion and influence. Proc. 16th ACM SIGKDD Int. Conf. Knowl. Discov. Data Min. - KDD '10 **5,** 1019–1028 (2010).

20. Nemhauser, G., Wolsey, L. & Fisher, M. An analysis of approximations for maximizing submodular set functions—I. Math. Program. (1978).

21. Alimi, T. O. et al. Prospects and recommendations for risk mapping to improve strategies for effective malaria vector control interventions in Latin America. Malar. J. **14,** 519 (2015).

22. Bhatt, S. et al. The effect of malaria control on Plasmodium falciparum in Africa between 2000 and 2015. Nature **526,** 207–211 (2015).

23. Kerlin, D. H. & Gatton, M. L. A simulation model of the within-host dynamics of Plasmodium vivax infection. Malar. J. **14,** 51 (2015).
